# Enhanced silver nanoparticle-induced pulmonary inflammation in a metabolic syndrome mouse model and resolvin D1 treatment

**DOI:** 10.1186/s12989-022-00495-6

**Published:** 2022-08-06

**Authors:** Saeed Alqahtani, Li Xia, Jonathan H. Shannahan

**Affiliations:** 1 School of Health Sciences, College of Health and Human Sciences, Purdue University, West Lafayette, IN USA; 2 Life Science and Environment Research Institute, King Abdulaziz City for Science and Technology (KACST), Riyadh, Saudi Arabia

**Keywords:** Nanoparticles (NPs), Inflammatory resolution, Specialized pro-resolving mediators (SPMs), Resolvin D1 (RvD1), Metabolic syndrome (MetS), Susceptibility, Omega-3 polyunsaturated fatty acids, Lipid supplementation, Chronic inflammation, Nanotoxicity, Failure of resolution

## Abstract

**Background:**

Metabolic syndrome (MetS) exacerbates susceptibility to inhalation exposures such as particulate air pollution, however, the mechanisms responsible remain unelucidated. Previously, we determined a MetS mouse model exhibited exacerbated pulmonary inflammation 24 h following AgNP exposure compared to a healthy mouse model. This enhanced response corresponded with reduction of distinct resolution mediators. We hypothesized silver nanoparticle (AgNP) exposure in MetS results in sustained pulmonary inflammation. Further, we hypothesized treatment with resolvin D1 (RvD1) will reduce exacerbations in AgNP-induced inflammation due to MetS.

**Results:**

To evaluate these hypotheses, healthy and MetS mouse models were exposed to vehicle (control) or AgNPs and a day later, treated with resolvin D1 (RvD1) or vehicle (control) via oropharyngeal aspiration. Pulmonary lung toxicity was evaluated at 3-, 7-, 14-, and 21-days following AgNP exposure. MetS mice exposed to AgNPs and receiving vehicle treatment, demonstrated exacerbated pulmonary inflammatory responses compared to healthy mice. In the AgNP exposed mice receiving RvD1, pulmonary inflammatory response in MetS was reduced to levels comparable to healthy mice exposed to AgNPs. This included decreases in neutrophil influx and inflammatory cytokines, as well as elevated anti-inflammatory cytokines.

**Conclusions:**

Inefficient resolution may contribute to enhancements in MetS susceptibility to AgNP exposure causing an increased pulmonary inflammatory response. Treatments utilizing specific resolution mediators may be beneficial to individuals suffering MetS following inhalation exposures.

**Supplementary Information:**

The online version contains supplementary material available at 10.1186/s12989-022-00495-6.

## Introduction

Metabolic syndrome (MetS) is increasingly prevalent in the U.S., where approximately 34% of individuals suffer from this health condition [1]. MetS is defined by having at least three of the following components (1) abdominal obesity, (2) hyperglycemia/insulin resistance, (3) hypertension, (4) reduced high-density lipoprotein (HDL), and (5) high triglycerides. Individuals with MetS have increased risk of chronic diseases including cancer, cardiovascular disease, type 2 diabetes and others [2, 3]. Epidemiological studies have reported individuals with MetS are sensitive to inhaled particulate matter (PM)-related health effects in comparison to healthy individuals [2, 4]. Specifically, distinct MetS components such as hypertension and excessive fat are associated with increased systemic inflammation markers such as C-reactive protein, white blood cells, and interleukin-6 following inhalation exposure to PM_2.5_ and PM_10_ [2, 5–8]. These findings support enhanced susceptibility to the inflammatory effects of inhaled particulate exposures due to MetS. Silver nanoparticles (AgNPs) have antimicrobial features resulting in their incorporation into a variety of consumer goods and biomedical applications resulting in numerous safety evaluations [9, 10]. Specifically, previous studies from our laboratory demonstrate exacerbated acute pulmonary inflammatory responses 24 h following AgNPs exposure in a MetS mouse model [11–13].

Lipids are dysregulated in MetS and are intricately involved in regulation of the immune system. Acute inflammatory signaling is initiated by induction of lipid mediators of inflammation (LMI). This proinflammatory state is resolved through subsequent signaling via specialized pro-resolving lipid mediators (SPMs). Disruption of these lipid signaling pathways can result in inefficient or failure of resolution causing chronic inflammation and disease progression [14, 15]. Inhalation exposures can modify pulmonary lipids involved in inflammatory signaling [16–18]. Recent studies from our laboratory determined reduced pulmonary eicosapentaenoic acid (EPA), docosapentaenoic acid (DHA), 18-hydroxy eicosapentaenoic acid (18-HEPE), 14-hydroxy docosahexaenoic acid (14-HDHA), 17-hydroxy docosahexaenoic acid (17-HDHA), maresin-1, and resolvin D1 (RvD1) levels as well as other SPMs 24 h after AgNP exposure in the MetS mouse model [11, 13]. Previously, preventative treatment with a combination of SPM precursors reduced ozone-induced inflammation demonstrating modification in SPM availability can influence pulmonary responses to inhaled exposures [17]. Our recent study determined the exacerbated pulmonary inflammatory response observed in a MetS mouse model 24 h following AgNP inhalation exposure was reduced by preventative treatment with distinct SPMs precursors, 14-HDHA and 17-HDHA while limited benefit resulted from 18-HEPE treatment [13]. Inefficiencies or failure in resolution may result in longer lasting inflammation, enhancing exposure-induced damage, and risk of inflammatory-driven diseases. Currently, there is a gap in our knowledge regarding sustained inflammation and the use of post-exposure SPM treatment to engage resolution signaling following NP exposures in MetS.

Resolvin D1 (RvD1) a final metabolite product of the ω-3 polyunsaturated fatty acid, DHA [13]. RvD1 is produced via lipoxygenase-mediated metabolism and promotes resolution by activation of G Protein-Coupled Receptor 32 (GPR32) or formyl peptide receptor 2 (ALX/FPR2) receptors [13]. The effectiveness of RvD1 as a treatment option has been investigated in various inflammatory diseases using a variety of models [19–23]. Specifically, RvD1 treatment reduced inflammation and improved lung function following challenges with lipopolysaccharides, cigarette smoke, bacteria, or hyperoxia [21, 22, 24–26]. Our current study, selected RvD1 for investigation based on our recent findings where RvD1 and its precursor 17-HDHA were specifically reduced in the MetS mouse model 24 h following AgNP exposure but unaltered in the healthy mouse model. Further, treatment with 17-HDHA prior to AgNP exposure reestablished pulmonary SPM levels in a mouse model of MetS and decreasing the pulmonary neutrophilic influx to levels similar to those observed in the healthy model [13]. These data suggest RvD1 may be a useful therapeutic strategy to address exacerbated exposure-induced health effects in MetS.

Individuals with MetS are susceptible to particulate exposures; however, mechanisms are unelucidated [8]. In our current study, we hypothesized AgNP exposure induces enhanced pulmonary inflammation and delayed resolution in a MetS mouse model compared to a healthy mouse model. Further, we hypothesized treatment with RvD1 could address deficiencies in SPMs we previously observed and reduce these exacerbated inflammatory responses. To address these hypotheses, pulmonary inflammatory endpoints were examined across a time course in healthy or MetS mouse models exposed AgNPs and subsequently treated with RvD1.

## Results

### Silver nanoparticle characterization

Citrate-coated 20 nm silver nanoparticles (AgNPs) were characterized to verify the manufacture specifications. AgNPs were determined to have a hydrodynamic size of 34.16 ± 0.43, polydispersion index of 0.37 ± 0.03, and ζ-potential of − 38.83 ± 0.25 in DI water at a concentration of 25 µg/mL. These parameters are all reported as mean ± standard deviation (*n* = 4) and are similar to manufacturer specifications as well as previous AgNPs used for toxicological evaluations [27–29].

### Mouse model characterization

**Fig. 1:**
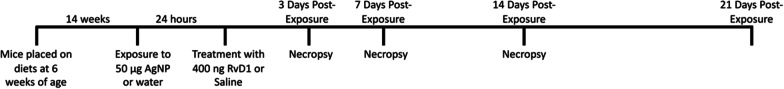
Experiment Design Timeline. Mice were fed a healthy or high-fat western diet for 14 weeks and exposed to either water (control) or AgNPs (50 µg) via oropharyngeal aspiration. 24 h post-exposure, mice were treated with saline (control) or RvD1 (400 ng) via oropharyngeal aspiration. Endpoints associated with inflammation and lipid metabolism were examined at 3-, 7-, 14-, and 21-days following AgNP exposure

Healthy and MetS mice were divided into controls or AgNP exposure groups, 24 h following exposure mice received vehicle or RvD1 treatment and samples were collected at 3-, 7-, 14-, and 21-days post AgNP-exposure (Fig. 1), To characterize the healthy and MetS mouse models, on either healthy or high-fat western (HFW) diets as well as the effects of exposure and treatment, markers of MetS including gained body weight (BW), serum total cholesterol (TC), high-density lipoprotein (HDL), low-density lipoprotein (LDL), and triacylglycerides (TG) were measured. HFW diet increased body weight compared to those fed the healthy diet (Additional file 1: Fig. S1A). Serum TC, HDL, and LDL were elevated in MetS mouse model compared the healthy model (Additional file 1: 1B–D). Serum TG did not differ between MetS or healthy mouse models due to diet (Additional file 1: Fig. S1E). Overall, these results were similar to our previous studies and others utilizing a diet-induced mouse model of MetS [11–13]. Previously, we demonstrated that the MetS mouse model also exhibits elevated plasma insulin level [12] while others have reported elevated blood glucose [30, 31] and hypertension [32]. AgNP exposure and/or treatment with RvD1 did not alter body weight or serum levels of TC, HDL, LDL, and TG in either healthy or MetS mouse models (Additional file 1: Fig. S1).

### BAL fluid markers of pulmonary inflammation

**Fig. 2:**
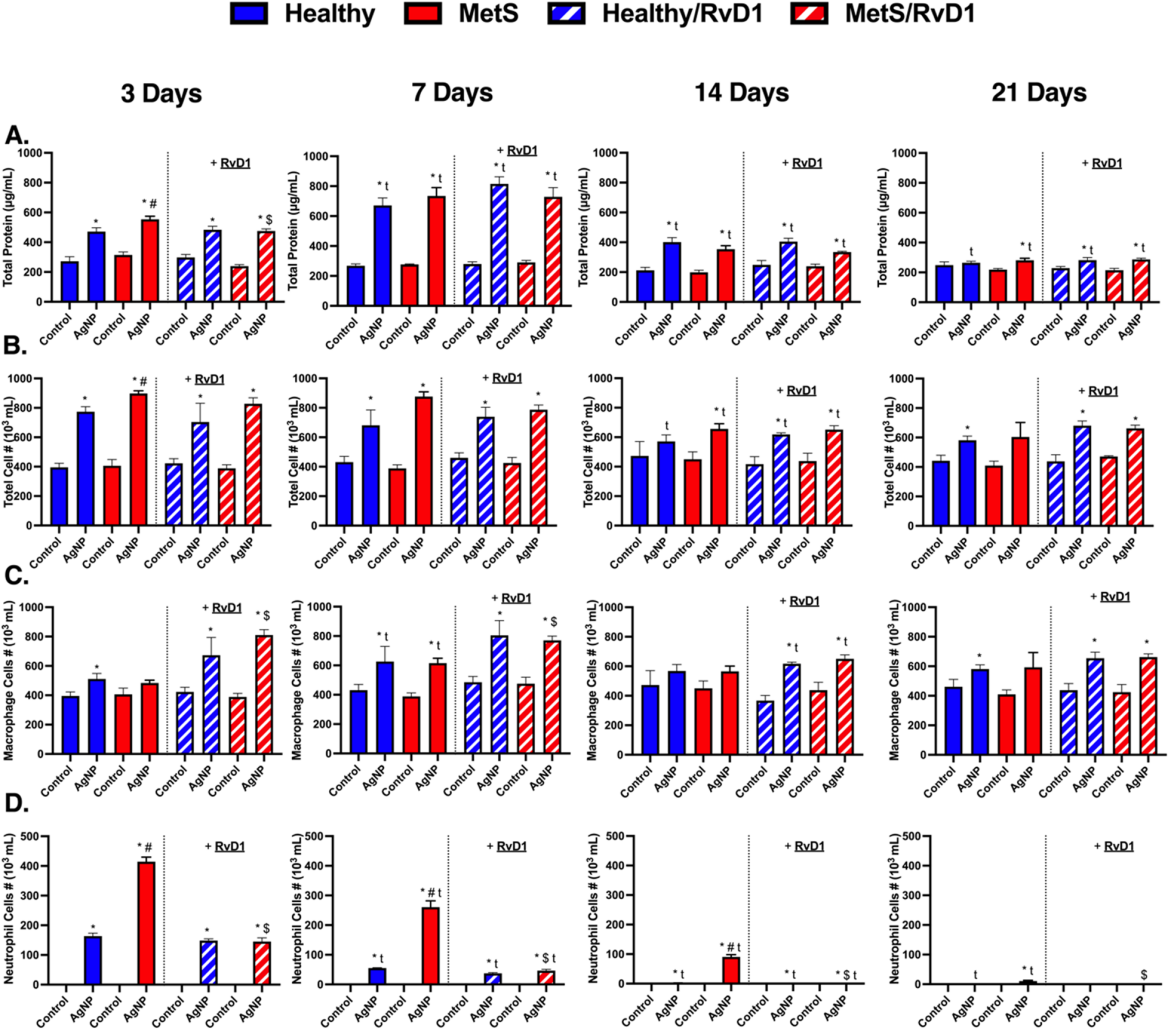
Chronic effect of AgNP exposure and modulation by RvD1 treatment on BALF **A** total protein, **B** total cell counts, **C** macrophage counts, and **D** neutrophil counts from healthy and MetS mice. 24 h following oropharyngeal aspiration of pharmaceutical grade sterile water (control) or AgNPs (50 µg) in sterile water, mice were oropharyngeal aspiration with 400 ng RvD1 or sterile saline (vehicle). Endpoints were evaluated at 3-, 7-, 14- and 21-days post AgNP exposures. Values are expressed as mean ± S.E.M. * AgNP exposure; # disease model; $ treatment; and t time point (p < 0.05)

Bronchoalveolar lavage (BAL) fluid parameters were examined to determine lung injury and pulmonary inflammatory responses. An increase in BAL fluid total protein concentrations is a marker of pulmonary permeability while alterations in cellular content were examined to assess inflammation. Total protein levels, total cell counts, macrophage counts, and neutrophil counts within BAL fluid were not altered at baseline between healthy and MetS controls (Fig. 2A–D). AgNP exposure elevated total protein levels in BAL fluid in both mouse models at 3, 7, and 14-days. At 21 days only AgNP exposed MetS mice demonstrated elevated total protein levels in BAL fluid. The MetS mouse model demonstrated exacerbated AgNP-induced increases in total protein levels compared to healthy mice at day 3 and 7. Total protein levels in BAL fluid were further increased in both exposed mouse models at day 7. The total protein levels in BAL fluid were demonstrated to decrease in both AgNP exposed mouse models at day 14 and 21 compared to the previous time points. RvD1 treatment only decreased BAL fluid total protein levels at day 3 in MetS exposed to AgNPs compared to MetS mice exposed to AgNPs and not receiving RvD1 (Fig. 2A). AgNP exposure elevated total cell influx in BAL fluid in both mouse models at 3 and 7 days, only in MetS mice at day 14, and only in healthy mice at day 21. The elevation in total cell counts was exacerbated in the MetS mouse model at day 3. Total cell counts in BAL fluid were decreased in exposed mouse models at day 14. RvD1 treatment did not alter the total cell counts in BAL fluid in either mouse model at any of the assessed time points (Fig. 2B). AgNP exposure elevated BAL fluid macrophage counts in healthy mice at 3-, 7-, and 21-days. AgNP exposure elevated BAL fluid macrophage counts in the MetS mouse model only at 7 days. Macrophage counts in BAL fluid were decreased at 14 days in both exposed mouse models receiving RvD1. RvD1 treatment elevated macrophages in BAL fluid in MetS exposed to AgNPs in comparison to exposed MetS mice not receiving RvD1 only at day 3 and 7 (Fig. 2C). AgNP exposure elevated neutrophil counts in BAL fluid in healthy mice at 3, 7, and 14 days while being elevated in MetS mice at all time points. Neutrophil influx in BAL fluid was exacerbated in exposed MetS at all time points. Neutrophil counts in BAL fluid decreased in exposed healthy mice not receiving RvD1 at day 7 compared to their matched groups (exposed healthy mice not receiving RvD1) at day 3. Additionally, neutrophil counts in BAL fluid decreased in exposed healthy mice not receiving RvD1 at day 14 and decreased in exposed MetS mice not receiving RvD1 at day 7. Furthermore, neutrophil counts continued to decrease in exposed MetS mice not receiving RvD1 at day 14 and day 21. RvD1 treatment decreased neutrophil counts in MetS exposed to AgNP in comparison to mice not receiving RvD1 and exposed to AgNPs at all time points (Fig. 2D).

### Hyperspectral analysis of internalized AgNPs

**Fig. 3:**
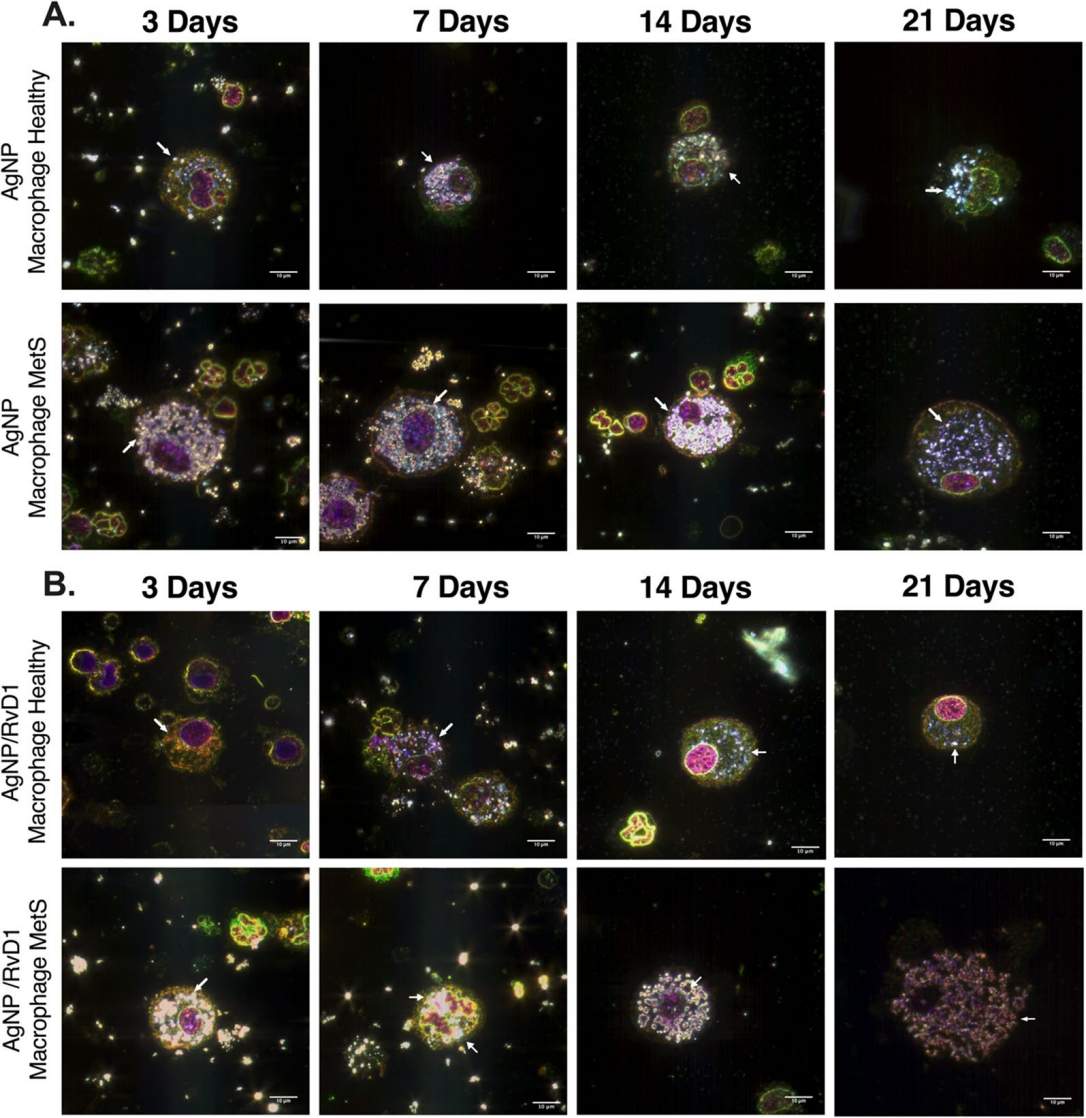
Hyperspectral analysis of AgNPs within macrophages collected from BAL fluid healthy and MetS mouse models. **A** Representative enhanced darkfield images of macrophages at 3, 7, 14, and 21 days following AgNP exposure at 50 µg from healthy and MetS mice not receiving RvD1 treatment. **B** Representative enhanced darkfield images of macrophages at 3, 7, 14, and 21 days following AgNP exposure from healthy and MetS mice receiving 400 ng RvD1 treatment. At least 1000 pixels of AgNPs were collected from mean spectra and then all spectra were normalized based on intensity for comparisons. White bar identifies 10 µm scaling. Representative images of internalized cells and spectral profiles of internalized AgNPs over the study time course can be found in Supplemental Fig. 2–4

Similar to previous publications, AgNPs were identified and characterized in collected BAL fluid immune cells via hyperspectral darkfield microscopy [13, 33, 34]. AgNPs were placed onto a clean slide and a mean spectral profile was generated to compare AgNPs associated with collected BAL cells from exposed mice. AgNPs were observed in macrophages in both exposed mouse models and associations were not modified due to RvD1 treatment (Fig. 3). Spectral mapping was utilized to confirm AgNPs within BAL cells (Additional file 1: Fig. S2). AgNPs were also observed in neutrophils in both exposed mouse models not receiving RvD1 at all time points (Additional file 1: Fig. S3A). Additionally, AgNPs were observed in neutrophils in both exposed mouse models receiving RvD1 at 3, 7, and 14 days (Additional file 1: Fig. S3B). Spectral profiles generated from internalized AgNPs demonstrated cell-specific shifts compared to the original AgNP spectrum (Additional file 1: Fig. S4). Specifically, internalization of AgNPs by macrophages and neutrophils in models resulted in distinct red shifts in the light-scattering spectrum compared to the original AgNP spectrum (Additional file 1: S4). No differences in AgNP spectral profiles were determined between models or due to treatment with RvD1 (Additional file 1: Fig. S4).

### Pulmonary gene expression analysis of pro-inflammatory markers

**Fig. 4:**
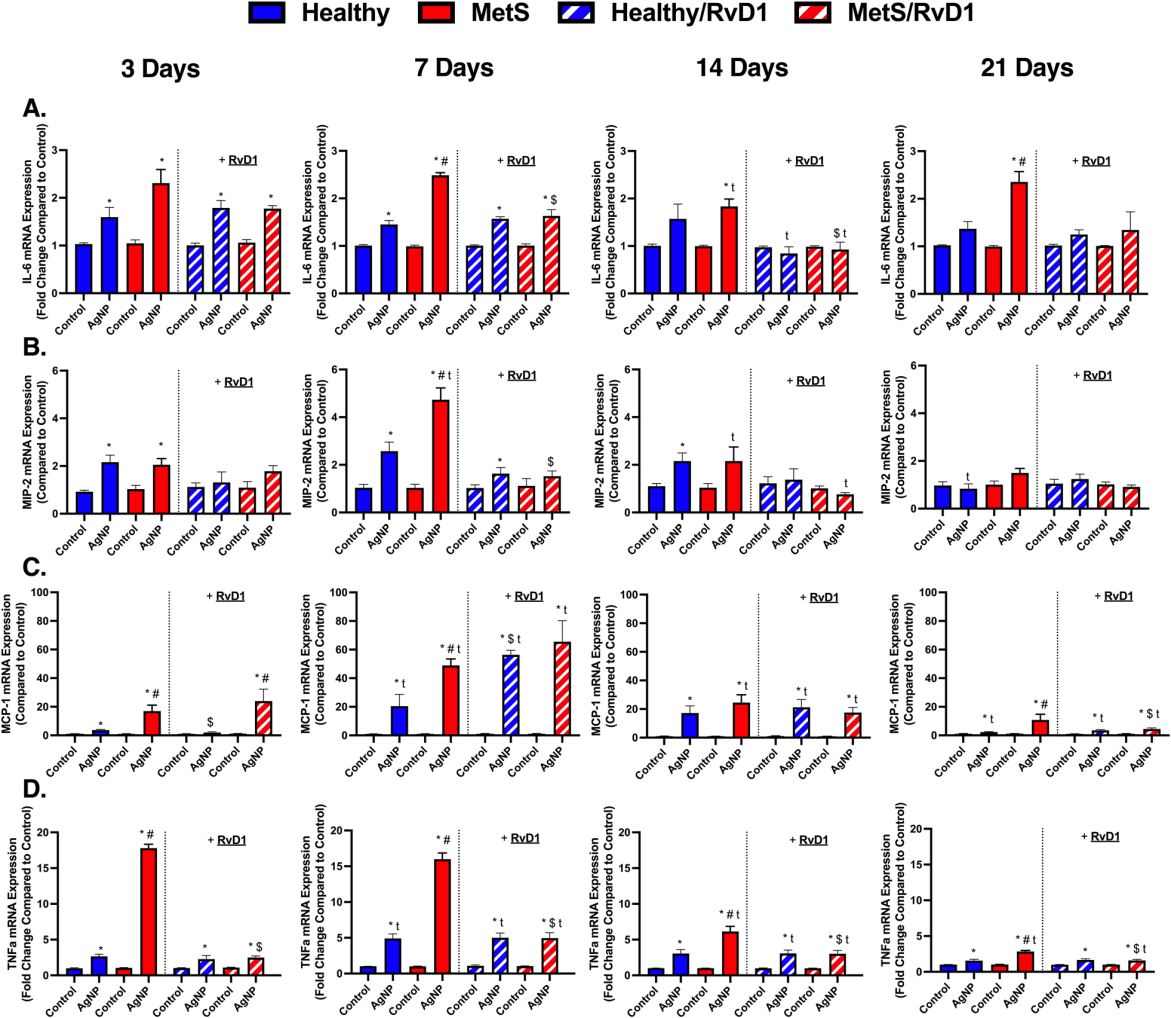
Chronic effect of AgNP exposure and modulation by RvD1 treatment on pulmonary inflammatory gene expression including **A** *Interleukin-6 (IL-6)*, **B** *macrophage inflammatory protein-2 (MIP-2)*, **C** *monocyte chemoattractant-1 (MCP-1),* and **D** *tumor necrosis factor alpha (TNF-α)* from healthy and MetS mice. 24 h following oropharyngeal aspiration of pharmaceutical grade sterile water (control) or AgNPs (50 µg) in sterile water, mice were oropharyngeal aspiration with 400 ng RvD1 or sterile saline (vehicle). Endpoints were evaluated at 3-, 7-, 14- and 21-days post AgNP exposures. Values are expressed as mean ± S.E.M. * AgNP exposure; # disease model; $ treatment; and t time point (p < 0.05)

Gene expression alterations within lung tissue samples were evaluated to determine AgNP-induced inflammation and modifications resulting from RvD1 treatment. Gene expression of *interleukin-6 (IL-6), macrophage inflammatory protein-2 (MIP-2), monocyte chemoattractant protein-1 (MCP-1),* and *tumor necrosis factor alpha (TNF-α)* were not changed between healthy and MetS models at baseline (Fig. 4A–D). AgNP exposure elevated *IL-6* in healthy mice at day 3 and 7. AgNP exposure elevated *IL-6* in MetS mice at all time points. This elevation in *IL-6* in exposed MetS mice was exacerbated compared to exposed healthy mice at day 7 and 21. *IL-6* decreased in both exposed mouse models receiving RvD1 at day 14. Treatment with RvD1 decreased *IL-6* in exposed MetS mice in comparison to exposed MetS mice not receiving RvD1 at day 7 and 14 (Fig. 4A). AgNP exposure elevated *MIP-2* in healthy mice at days 3, 7, and 14 while only elevating *MIP-2* in MetS mice at days 3 and 7. This elevation in *MIP-2* was exacerbated in exposed MetS mice at day 7 compared to healthy. *MIP-2* was elevated in exposed MetS mice not receiving RvD1 at day 7 compared to day 3. This elevation in *MIP-2* in exposed MetS mice not receiving RvD1 decreased at day 14 and continued to decrease at day 21. Treatment with RvD1 decreased *MIP-2* in exposed MetS mice at day 7 (Fig. 4B). AgNP exposure elevated *MCP-1* in both mouse models at all time points. The elevation in *MCP-1* in exposed MetS mice was exacerbated compared to exposed healthy mice at day 3, 7, and 21. *MCP-1* was elevated in exposed MetS mice not receiving RvD1 at day 7 and decreased at day 14. *MCP-1* decreased in exposed healthy mice not receiving RvD1 at day 21. *MCP-1* was elevated in exposed MetS mice receiving RvD1 at day 7 and was reduced by day 14. *MCP-1* further decreased in both exposed models receiving RvD1 at day 21. Treatment with RvD1 decreased *MCP-1* in exposed healthy mice at day 3. RvD1 treatment elevated *MCP-1* in exposed healthy mice at day 7 compared to exposed healthy mice not receiving RvD1. RvD1 decreased *MCP-1* in exposed MetS mice in comparison to exposed MetS not receiving RvD1 at day 21 (Fig. 4C). AgNP exposure elevated *TNF-α* in both models at all time points. The elevation in *TNF-α* was exacerbated in MetS mice at all time points. *TNF-α* was elevated in exposed healthy mice not receiving RvD1 at day 7 compared to day 3. *TNF-α* was decreased in exposed MetS mice not receiving RvD1 at day 14 and further at day 21. *TNF-α* was elevated in both exposed models receiving RvD1 at day 7 and was decreased at day 14 as well as day 21. RvD1 treatment decreased *TNF-α* in exposed MetS mice compared to exposed MetS mice not receiving RvD1 at all time points (Fig. 4D).

### Pulmonary gene expression analysis of anti-inflammatory markers

**Fig. 5:**
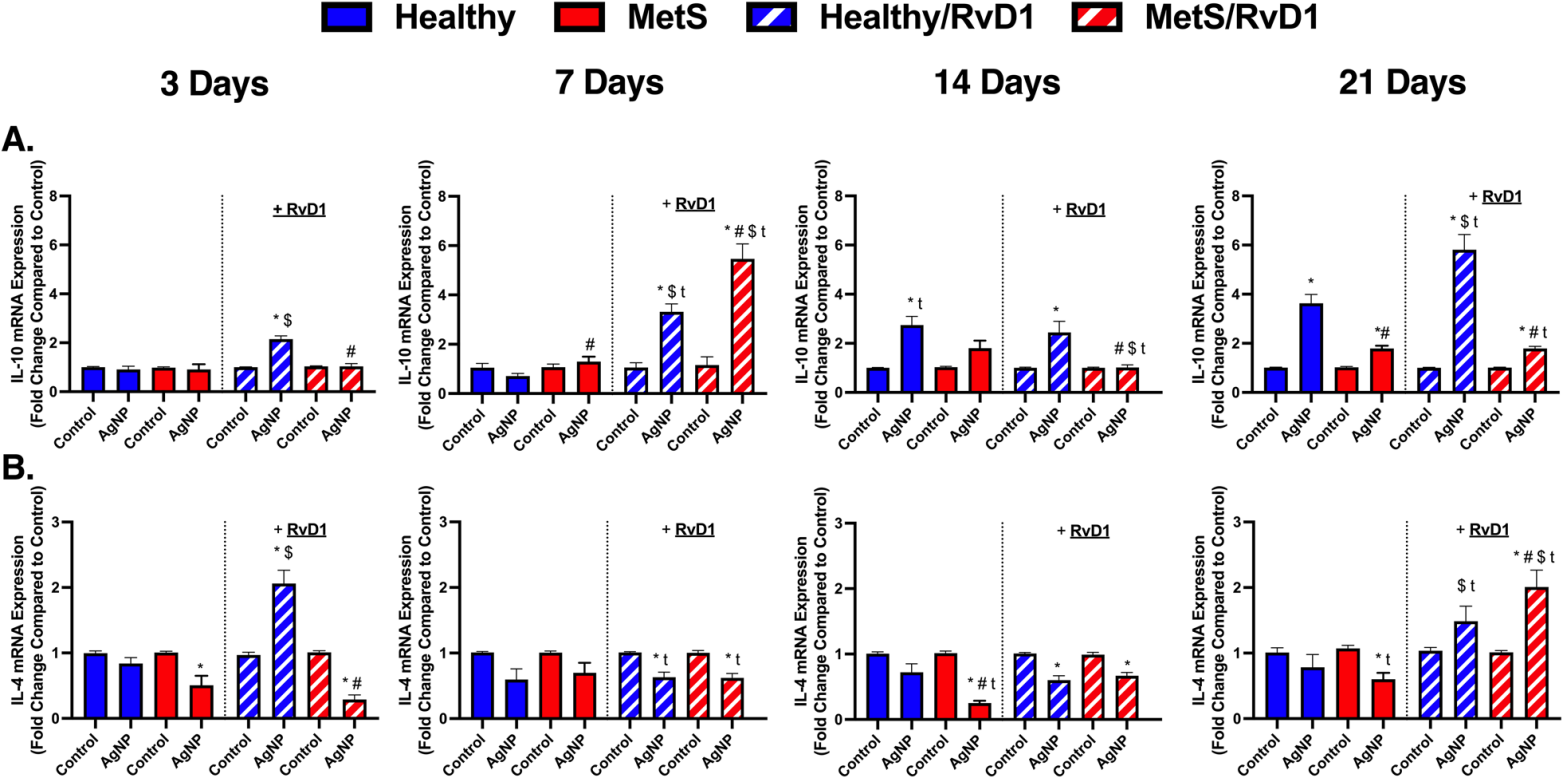
Chronic effect of AgNP exposure and modulation by RvD1 treatment on pulmonary anti-inflammatory gene expression including **A** *Interleukin-10 (IL-10)* and **B** *Interleukin-4 (IL-4)* from healthy and MetS mice. 24 h following oropharyngeal aspiration of pharmaceutical grade sterile water (control) or AgNPs (50 µg) in sterile water, mice were oropharyngeal aspiration with 400 ng RvD1 or sterile saline (vehicle). Endpoints were evaluated at 3-, 7-, 14- and 21-days post AgNP exposures. Values are expressed as mean ± S.E.M. * AgNP exposure; # disease model; $ treatment; and t time point (p < 0.05)

Expression levels of genes involved in resolution within lung tissue samples were evaluated to determine differences due to disease, time, and RvD1 treatment in the AgNP-induced inflammatory response. Gene expression of *interleukin-10 (IL-10),* and *interleukin-4 (IL-4)* were not changed between healthy and MetS at baseline. AgNP exposure elevated *IL-10* in MetS mice not receiving RvD1 treatment in comparison to exposed healthy mice not receiving RvD1 at day 7 (Fig. 5A). AgNP exposure elevated *IL-10* in healthy mice not receiving RvD1 at day 14 and 21. AgNP exposure elevated *IL-10* in MetS mice not receiving RvD1 at day 21. RvD1 treatment elevated *IL-10* in healthy mice exposed to AgNPs at all time points. AgNP exposure elevated *IL-10* in MetS mice receiving RvD1 at day 7 and 21. The elevation in *IL-10* was enhanced in exposed MetS receiving RvD1 compared to exposed healthy mice receiving RvD1 at day 7. The elevation in *IL-10* was decreased in exposed MetS receiving RvD1 compared to exposed healthy mice receiving RvD1 at day 21. RvD1 treatment elevated *IL-10* in exposed healthy mice compared to exposed healthy mice not receiving RvD1 at day 3. RvD1 treatment elevated *IL-10* in both exposed mouse models at day 7. The treatment with RvD1 treatment decreased *IL-10* in exposed MetS mice compared to exposed MetS mice not receiving RvD1 at day 14. The treatment with RvD1 treatment increased *IL-10* again in exposed healthy mice compared to exposed healthy mice not receiving RvD1 at day 21. *IL-1*0 elevated in both exposed mouse models receiving RvD1 at day 7 compared to their matched mice at day 3. *IL-10* elevated in exposed healthy mice not receiving RvD1 at day 14. *IL-10* decreased in exposed MetS mice receiving RvD1 at day 14. *IL-10* was elevated in both exposed mouse models receiving RvD1 at day 21 (Fig. 5A).

AgNP exposure decreased *IL-4* in MetS mice not receiving RvD1 treatment at day 7, 14, and 21 (Fig. 5B). This reduction in *IL-4* in exposed MetS mice was exacerbated compared to exposed healthy mice not receiving RvD1 at day 14. AgNP exposure elevated *IL-4* in healthy mice receiving RvD1 at day 3 and 21. AgNP exposure decreased *IL-4* in healthy mice receiving RvD1 at day 7 and 14. AgNP exposure decreased *IL-4* in MetS mice receiving RvD1 at day 3, 7 and 14. The reduction in *IL-4* in exposed MetS mice receiving RvD1 was exacerbated compared to exposed healthy mice receiving RvD1 at day 3. AgNP exposure elevated *IL-4* in MetS mice receiving RvD1 at day 21. *IL-4* decreased in exposed healthy mice receiving RvD1 at day 7. *IL-4* expression was elevated in exposed MetS mice receiving RvD1 at day 7. Additionally, *IL-4* gene was elevated in both exposed mouse models receiving RvD1 at day 21 (Fig. 5B).

### Pulmonary gene expression analysis of lipid metabolism

**Fig. 6:**
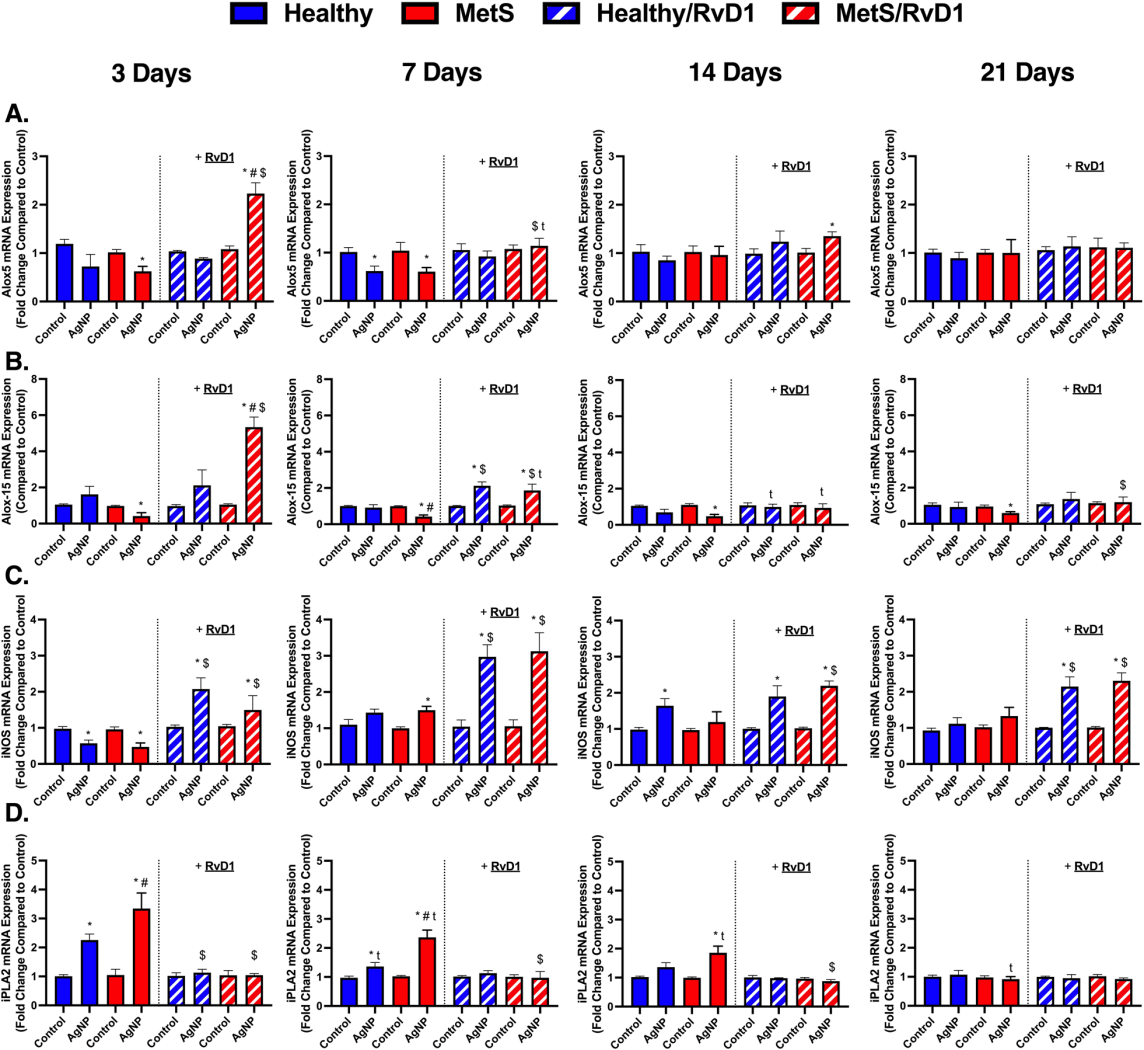
Chronic effect of AgNP exposure and modulation by RvD1 treatment on pulmonary gene expression associated with lipid metabolism including **A** *Arachidonate 5-lipoxygenase (ALOX-5),* **B** *arachidonate 15-lipoxygenase (ALOX-15),* **C** *inducible nitric oxide synthase (iNOS), and* **D** *phospholipase A2 (iPLA2)* from healthy and MetS mice. 24 h following oropharyngeal aspiration of pharmaceutical grade sterile water (control) or AgNPs (50 µg) in sterile water, mice were oropharyngeal aspiration with 400 ng RvD1 or sterile saline (vehicle). Endpoints were evaluated at 3-, 7-, 14- and 21-days post AgNP exposures. Values are expressed as mean ± S.E.M. * AgNP exposure; # disease model; $ treatment; and t time point (p < 0.05)

Lipid metabolism gene expression levels within lung tissue samples were evaluated to determine differences due to MetS, time course, and RvD1 treatment following AgNP-induced inflammation. Gene expression of *arachidonate 5-lipoxygenase (ALOX-5)*, *arachidonate 15-lipoxygenase (ALOX-15), inducible nitric oxide synthase (iNOS),* and *phospholipase A2 (iPLA2)* were not changed between healthy and MetS mice at baseline. AgNP exposure decreased the gene expression of *ALOX-5* in exposed MetS mice not receiving RvD1 treatment at day 3 (Fig. 6A) and in both mouse models at day 7. AgNP exposure increased *ALOX-5* in MetS mice receiving RvD1 at day 3 and 14 and this elevation was exacerbated in MetS mice compared to healthy at day 3. *ALOX-5* gene expression was decreased in exposed MetS mice receiving RvD1 at day 7. RvD1 treatment elevated *ALOX-5* in exposed MetS compared to exposed MetS mice not receiving RvD1 at day 3 and 7 (Fig. 6A). AgNP exposure did not alter *ALOX-15* in healthy mice at any of the evaluated time points (Fig. 6B). AgNP exposure decreased *ALOX-15* in exposed MetS mice not receiving RvD1 at all time points, and this reduction was exacerbated in MetS mice not receiving RvD1 at day 7. AgNP exposure elevated *ALOX-15* in MetS mice receiving RvD1 at day 3 and 7 and in healthy mice receiving RvD1 at day 7. *ALOX-15* was decreased in both exposed mouse models receiving RvD1 at day 14. RvD1 treatment elevated *ALOX-15* in exposed MetS compared to exposed healthy mice receiving RvD1 at day 3. RvD1 treatment elevated *ALOX-15* in both exposed mouse models in comparison to exposed mouse models not receiving RvD1 at day 7 and in MetS mice at day 21 (Fig. 6B). AgNP exposure decreased the gene expression of *iNOS* in both exposed mouse models not receiving RvD1 at day 3 (Fig. 6C). AgNP exposure increased *iNOS* in exposed MetS mice not receiving RvD1 at day 7. AgNP exposure increased *iNOS* in exposed healthy mice not receiving RvD1 at day 14. RvD1 treatment elevated *iNOS* in both exposed models at days 3, 7, and 21 while also elevating *iNOS* in exposed MetS at day 14 (Fig. 6C). AgNP exposure elevated *iPLA2* in both models not receiving RvD1 at day 3 and 7, and this elevation was exacerbated in MetS (Fig. 6D). AgNP exposure elevated *iPLA2* in exposed MetS mice not receiving RvD1 at day 14. *iPLA2* was decreased in both models not receiving RvD1 treatment at day 7. *iPLA2* decreased in exposed MetS not receiving RvD1 at day 14 and further at day 21. RvD1 treatment decreased *iPLA2* in both models compared to exposed models not receiving RvD1 at day 3. RvD1 treatment decreased *iPLA2* in exposed MetS mice compared to exposed MetS mice not receiving RvD1 at day 7 and 14 (Fig. 6D).

### Inflammatory chemokines/cytokines

**Fig. 7:**
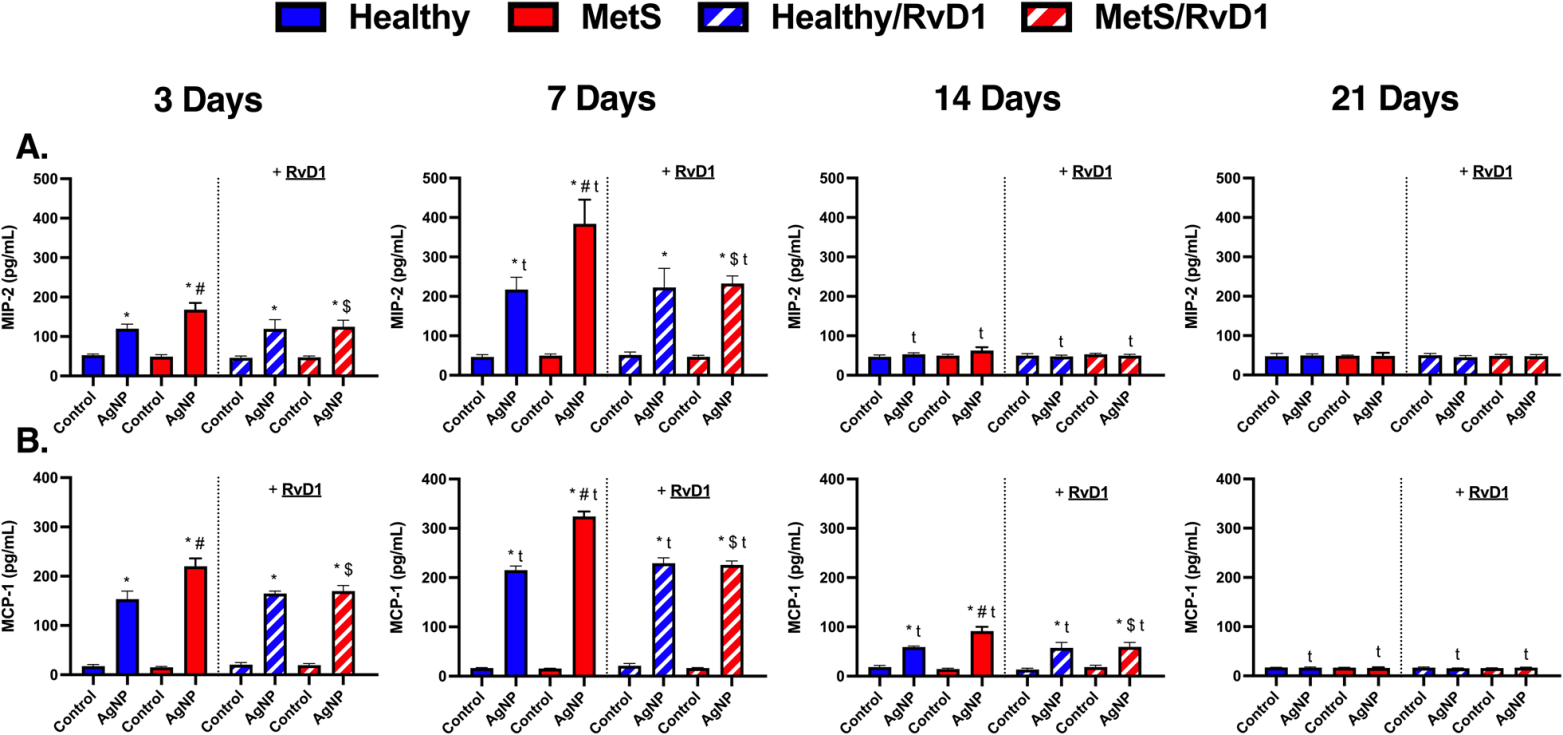
Chronic effect of AgNP exposure and modulation by RvD1 treatment on pulmonary inflammatory protein level including **A** macrophage inflammatory protein-2 and **B** monocyte chemoattractant-1 protein levels from BAL fluid healthy and MetS mice. 24 h following oropharyngeal aspiration of pharmaceutical grade sterile water (control) or AgNPs (50 µg) in sterile water, mice were oropharyngeal aspiration with 400 ng RvD1 or sterile saline (vehicle). Endpoints were evaluated at 3-, 7-, 14- and 21-days post AgNP exposures. Values are expressed as mean ± S.E.M. * AgNP exposure; # disease model; $ treatment; and t time point (p < 0.05)

**Fig. 8:**
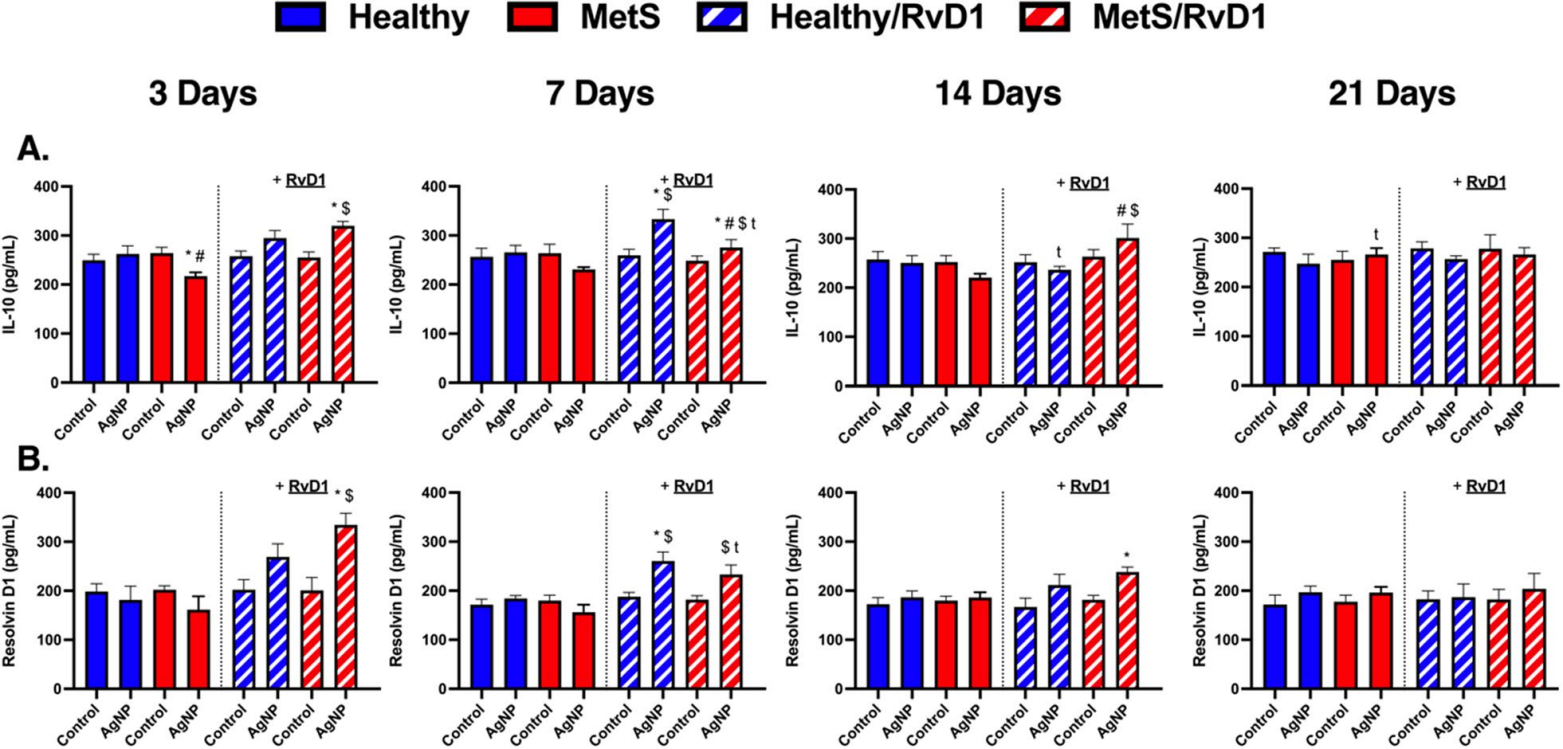
Chronic effect of AgNP exposure and modulation by RvD1 treatment on pulmonary pro-inflammatory resolution protein level **A** Interleukin 10 protein and on lipid involved in inflammatory resolution **B** Resolvin D1 levels from BAL fluid healthy and MetS mice. 24 h following oropharyngeal aspiration of pharmaceutical grade sterile water (control) or AgNPs (50 µg) in sterile water, mice were oropharyngeal aspiration with 400 ng RvD1 or sterile saline (vehicle). Endpoints were evaluated at 3-, 7-, 14- and 21-days post AgNP exposures. Values are expressed as mean ± S.E.M. * AgNP exposure; # disease model; $ treatment; and t time point (p < 0.05)

To further evaluate differences due to disease, time, and RvD1 treatment following AgNP-induced pulmonary inflammation, chemokines/cytokines protein and RvD1 lipid levels were measured within collected BAL fluid samples. Concentration levels of macrophage inflammatory protein 2 (MIP-2) and monocyte chemoattractant 1 (MCP-1), interleukin-10 (IL-10), and RvD1 were not changed between models at baseline at any evaluated time points. AgNP exposure elevated the concentration levels of MIP-2 in both exposed models not receiving RvD1 treatment at days 3 and 7, and this induction was exacerbated in MetS (Fig. 7A). AgNP exposure increased MIP-2 level in both exposed models receiving RvD1 at days 3 and 7, and this elevation was exacerbated in MetS at day 7. MIP-2 was elevated in exposed MetS mice receiving RvD1 following AgNP exposure at day 7 compared to their matched groups at day 3. MIP-2 levels were decreased in both exposed models receiving or not receiving RvD1 at day 14. RvD1 treatment decreased MIP-2 level in exposed MetS mice in comparison to exposed MetS not receiving RvD1 at day 3 and 7 (Fig. 7A). AgNP exposure elevated MCP-1 in both mouse models not receiving RvD1 at days 3, 7, and 14, and this elevation was exacerbated in MetS (Fig. 7B). AgNP exposure increased MCP-1 level in both models receiving RvD1 at days 3, 7 and 14. MCP-1 levels were elevated in both exposed mouse models receiving and in not receiving RvD1 following AgNP exposure at day 7 compared to day 3. MCP-1 levels decreased in all exposed mouse models receiving or not receiving RvD1 at day 14, and further reduced at day 21. RvD1 treatment decreased MCP-1 levels in exposed MetS mice at day 3, 7, and 14 (Fig. 7B). AgNP exposure decreased IL-10 in exposed MetS mice not receiving RvD1 treatment at days 3, and this reduction was exacerbated in MetS (Fig. 8A). AgNP exposure increased IL-10 levels in both exposed mouse models receiving RvD1 at day 7, and this elevation was exacerbated in healthy. AgNP exposure increased IL-10 level in exposed MetS mice receiving RvD1 at day 14 and this elevation was exacerbated compared to exposed healthy mice receiving RvD1 at day 14. IL-10 levels decreased in exposed MetS mice receiving RvD1 at day 7 day compared to in exposed MetS mice receiving RvD1 at day 3. IL-10 levels decreased in exposed healthy mice receiving RvD1 at day 14. IL-10 concentration level increased in exposed MetS mice not receiving RvD1 at day 21. RvD1 treatment elevated IL-10 level in exposed MetS mice compared to exposed MetS mice not receiving RvD1 at day 3, 7, and 14, and this elevation was exacerbated in MetS. Treatment with RvD1 elevated IL-10 levels in exposed healthy mice at day 7 (Fig. 8A). MetS mice exposed to AgNPs and treated with RvD1 demonstrated elevated BAL fluid RvD1 at days 3, 7, and 14. Healthy mice exposed to AgNPs demonstrated elevated levels of RvD1 at day 7. RvD1 was reduced in exposed MetS mice receiving RvD1 at day 7 compared to exposed MetS mice receiving RvD1 at day 3 (Fig. 8B).

## Discussion

Individuals with MetS are sensitive to inhaled exposures, however, the mechanisms responsible remain unelucidated [2, 4, 35]. Lipids are dysregulated in MetS and are intricately involved in the activation and resolution of inflammation. Recently, we demonstrated via lipid screening and targeted mass spectrometry-based approaches, SPMs which mediate inflammatory resolution were acutely disrupted 24 h following AgNP exposure in a MetS mouse model compared to healthy [11, 13]. This disease-related dysregulation of lipids may contribute to exacerbated and sustained pulmonary inflammation following particulate inhalation. Further, reductions in specific resolution mediators due to MetS, suggested by our previous studies, may be potential treatments to regulate exposure-induced inflammation. In the current study, we hypothesized AgNP exposure would result in exacerbated and prolonged inflammation in MetS and RvD1 could be used as a treatment. To test this hypothesis, healthy and MetS mouse models were exposed to water (control) or AgNPs to initiate an inflammatory response and 24 h later were treated with either saline (treatment control) or RvD1. At selected time points (3-, 7-, 14-, and 21-days post AgNP exposure) endpoints of inflammation and lipid metabolism were assessed. Consistent with our previous and the findings of others, AgNP exposure induced markers of pulmonary inflammation in both mouse models [11–13, 36, 37]. AgNP-induced inflammation was determined to be enhanced in the MetS mouse model compared to healthy at the time points evaluated, while RvD1 reduced AgNP-induced changes in inflammatory endpoints in MetS mice. Overall, these findings suggest enhanced inflammation and delayed resolution in a MetS mouse model which could contribute to enhanced susceptibility. Additionally, our findings demonstrate potential treatment strategies targeting resolution signaling could counteract exacerbated inflammatory responses in the susceptible population.

The primary route of AgNP exposure is inhalation where they cause sustained pulmonary toxicity and inflammation. Specifically, a single exposure to a AgNP similar to those utilized in our current study and at the same concentration, caused inflammation consisting of pulmonary neutrophilic influx, induction of inflammatory cytokines and chemokines, and oxidative stress lasting for 21 days in healthy rodent models [38, 39]. There is a gap in our knowledge regarding AgNP-induced pulmonary toxicity in prevalent disease conditions. Recently, we demonstrated enhancement of AgNP-induced acute inflammatory markers in a MetS mouse model corresponding with deficits in pulmonary SPMs at 24 h post-exposure [13]. The disruption in SPMs in MetS and the exacerbated inflammatory markers were inhibited by pre-treatment with distinct SPM precursors (14-HDHA or 17-HDHA) [13]. However, SPM precursor treatment was not observed to alter the neutrophilic influx observed in the exposed healthy mouse model. These findings suggested dysregulation of SPMs contribute to the enhanced susceptibility observed in the MetS mouse model acutely following AgNP exposure. Our current study demonstrates sustained exacerbations in inflammatory markers in a MetS mouse model compared to healthy.

Chronic inflammation, a result of failure of resolve, has been linked to progression of cardiovascular disease, diabetes mellitus, autoimmune conditions, cancer, and other diseases [40]. Treatments countering specific decreases in SPMs observed in our previous studies at 24 h post-exposure may be useful in addressing susceptibility to exposures in MetS [11, 13]. In our current study, the selected SPM, RvD1, is derived from metabolism of DHA [41, 42]. Previously, dietary supplementation of DHA (13 weeks) prevented systemic lupus erythematosus, by suppressing recruitment of immune cells to the lung, induced by silica exposure in a female NZBWFI mouse model [43]. Additionally, pretreatment with a cocktail of DHA intermediary metabolites (14-HDHA, 17-H-DHA, and PDX) reduced ozone-caused lung inflammation in a healthy animal model [17]. These studies demonstrate treatments with precursors to increase DHA-derived SPMs can reduce the pulmonary inflammatory response following exposures. Treatment with distinct SPMs (RvD1 and RvD2) have also demonstrated efficacy in reducing inflammatory disease triggered by *E. coli* infection, hyperoxia, or tobacco smoke [42, 44–46]. Specifically, RvD1 attenuated emphysema and sustained inflammation induced by cigarette smoke in C57BL/6 mice [23]. Our current study supports these previous findings and specifically demonstrated RvD1 could be utilized following the establishment of inflammation as a treatment to address the enhanced inflammatory response associated with MetS. Specifically, the MetS mouse model demonstrated increased-neutrophil influx at all time points assessed through 21 days post-exposure which was reduced to levels observed in the healthy mouse model by RvD1 treatment. Further, treatment with RvD1 inhibited AgNP-induced increases of MIP-2, IL-6, MCP-1, and TNF-α in MetS mice to levels observed in healthy over the time course. These findings suggest these inflammatory mediators contribute to the enhanced MetS inflammatory response and are sensitive to RvD1 regulation. The inhibition of these inflammatory markers by RvD1 was consistent with other studies. Specifically, RvD1 was found to reduce TNF-α, IL-6, MIP-2, MCP-1 in both in vitro and in vivo models following LPS and ischemia–reperfusion challenges [20–22, 47, 48]. These alterations in inflammatory cytokines/chemokines by RvD1 are associated with inhibition of NF-κB and C/EBP activation in lung tissue as well as decreases in BAL fluid levels of IL-6 in inflamed lungs mediated by the IgG immune complex [49]. Interestingly, RvD1 treatment did not affect basal levels of inflammatory gene expression in our study at the time points assessed. Previous studies have demonstrated a similar lack of basal modifications due to RvD1 treatment. An investigation of the ability of RvD1 to treat cigarette smoke-induced inflammation in mice demonstrated no basal alterations in lavage fluid neutrophils or lung *IL-6, MCP-1, TNF-α,* and *CXCL-1* gene expression levels in unexposed controls [23]. However, mice exposed to cigarette smoke did demonstrate reductions following RvD1 treatment, although not below untreated control levels.

iNOS regulates inflammation via modulation of TNF-α, and IL-6 production [50]. MCP-1 is reported to suppress *iNOS,* enhancing lung inflammation in mice following bacterial lipopolysaccharide (LPS) challenge [51]. Further, elevation in *iNOS* gene expression is linked to the suppression of M1 macrophage polarization [50]. Other exposures have demonstrated modulation of iNOS activity, suggesting dysregulation in iNOS may contribute to induced-sustained inflammatory responses to exposures [52, 53]. These studies suggest reduction in *iNOS* observed in MetS following AgNP exposure may contribute to exacerbated inflammation. IL-10 and IL-4 have anti-inflammatory effects via inhibition of proinflammatory cytokines/chemokines expression and enhancing the clearance of PMNs thereby reducing inflammation [54, 55]. *IL-10* and *IL-4* gene expression were elevated in the healthy mouse model at 7 days post-exposure whereas no increases were observed in the MetS mouse model until 21 days post-exposure. This delay in the induction of these anti-inflammatory genes in MetS suggest potential roles of diminished IL-10 and IL-4 production in the sustained inflammatory response following AgNP exposure. Studies demonstrate that RvD1 inhibits IL-6, IL-8, TNF-α without affecting IL-10 and promotes IL-10 targets such as heme oxygenase-1 (HO-1) in inflamed human adipose tissues [56–58]. RvD1 treatment increased production of IL-4 and IL-10 earlier in our time course in MetS suggesting an enhancement of anti-inflammatory signaling. Moreover, RvD1 enhances macrophage recruitment, which was observed in our study, and specifically recruits M2-like IL-10 producing macrophages [19, 20, 59].

Inflammatory processes including activation and resolution, are active and coordinated processes essential for the immune system to respond to a variety of challenges (i.e., exposures, pathogens, etc.). Inappropriate inflammatory responses can be detrimental to health. For example, exacerbated inflammation, a result of failure of resolution, can result in tissue damage and the development of diseases while suppression of inflammatory responses can enhance susceptibility to pathogens and reduce clearance of foreign materials [60, 61]. In our current study, RvD1 inhibited the exacerbated neutrophil influx in a MetS mouse model in response to AgNPs to a level comparable to that observed in the healthy mouse model. Therefore, RvD1 appears to reestablish the healthy or normal response to AgNP exposure in the MetS mouse via inhibition of specific inflammatory signaling. RvD1 limited the proinflammatory MIP-2, MCP-1, and IL-6 protein levels in MetS mice following AgNP exposure. Our previous investigations of atorvastatin and SPM precursors (14HDHA and 17HDHA) determined reduced inflammatory signaling mediators (MIP-2, MCP-1, and IL-6) in both exposed healthy and MetS [11, 13]. These findings suggest treatment with specific SPMs, such as RvD1, could address exacerbated inflammation observed in MetS while other SPM treatments may be useful in broader populations.

The ω-3 fatty acid, DHA is embedded in cellular membranes and is cleaved by iPLA2. Lipoxygenases metabolize DHA into 14-HDHA and 17-HDHA substrates and then to the SPMs, neuroprotectin 1 (NPD1) and the resolvin D series, which signal for increased cell survival, and inflammatory resolution [62, 63]. RvD1 is the final metabolite product of 17-HDHA, and signals via GPR32 and ALX/FPR2, to promote resolution [13, 64]. Previously, the impact of ω-3 fatty acids themselves and their metabolites have been evaluated in numerous investigations [13, 17, 43, 65]. These examinations emphasized the potential benefits of lipid supplementation of ω-3 fatty acids to enhance SPM levels. Alterations in enzymes such as lipoxygenases (LOX-5/15)-involved in SPMs production, may contribute to exacerbated inflammatory responses in MetS. Inhalation exposure to ozone decreases pulmonary *LOX-5/15* in a mouse model [17]. AgNP exposure was determined to decrease *ALOX-5* and *ALOX-15* mRNA gene expression in MetS mice suggesting alterations in metabolic processes mediating SPM production. RvD1 was found to inhibit the observed AgNP-induced alterations in lipoxygenase mRNA gene expression. This is consistent with a 17-HDHA treatment, a precursor of RvD1, that was found to increase SPM levels in the MetS mouse lung tissue while also decreasing the lipoxygenase gene expressions [13, 17]. RvD1 was found to alter ALOX-5 localization, which suggests that RvD1 can modify ALOX activity [66, 67]. Together, these findings suggest RvD1 treatment may reestablish lipoxygenases activity in MetS mice, allowing for production of SPMs, decreasing inflammatory processing following inhalation exposure [68]. Resolvins are ligands acting on resolution receptors, signaling to inhibit proinflammatory, initiate resolution, and facilities repair processes [69–73]. Our previous study demonstrated baseline reductions in resolution receptor protein expression in MetS mice that may contribute to susceptibility. Additionally, resolution receptors were reduced following AgNP exposures in both mouse models suggesting NP exposures can impair resolution signaling [13]. RvD1 treatment following AgNP exposure reduced inflammatory markers which was consistent with our previous study evaluating pre-treatment with SPM precursors [13]. This suggests ligand availability is likely a limiting factor in resolution capacity within MetS mice following AgNP exposure. Overall, supplementation with SPMs such as RvD1 could be used as a treatment for specific susceptible groups.

Our assessment is of value as it demonstrates alterations in pulmonary inflammatory endpoints due to an underlying disease condition and suggests a treatment, however, there are limitations present. Specifically, it is difficult to fully manifest and characterize human MetS in a mouse model. For example, mice lack the cholesterol ester protein which facilities the movement of cholesterol from HDL to LDL and triglycerides in humans [74]. In humans, this protein increases the prevalence of LDL and triglycerides which are key components of MetS. Without this protein, mice are predisposed to higher HDL levels and lower levels of LDL and triglycerides than humans on high fat content diets. However, even with these deficiencies, the model we utilized is the most accepted rodent model to evaluate MetS as it replicates key components of the disease [30, 32, 75, 76]. Another limitation of the study is that we utilized a single relatively high dose of AgNPs to initiate inflammation. This dose was utilized to replicate previous examinations and was necessary to induce an inflammatory response that could be examined for variations due to disease and RvD1 treatment [11–13, 27, 36, 77]. Future studies would benefit from lower doses and/or repeated exposures to investigate susceptibility and inflammatory signaling. The use of RvD1 allowed for the examination of a specific resolution treatment in a susceptible MetS mouse model. RvD1 was selected based on our previous investigations, however, deficits in other SPMs such as maresin-1 and lipoxins were also previously determined and could be applied to the MetS mouse model following exposures as treatments. Additionally, it is likely that failure to resolve inflammation one of multiple contributors to susceptibility. The earliest time point we have previously investigated has been 24 h after exposure, it is therefore likely differential proinflammatory lipids are induced at earlier time points following AgNP exposure and require investigation. Future studies are necessary to examine earlier time points during inflammatory induction where pro-inflammatory lipid mediators and other signaling processes may be exacerbated. Lastly, future studies need to investigate sex-related variations which may contribute to susceptibility as well as dysregulation of SPM metabolism and clearance.

In conclusion, MetS is associated with enhanced susceptibility to inhalation exposure-induced inflammation and toxicity. In a mouse model of MetS, we determined inflammation was elevated in comparison to a healthy model through 21 days after AgNP exposure and RvD1 treatment could decrease alterations in inflammatory markers. Overall, strategies to reestablish SPM-mediated resolution signaling may be beneficial to individuals with MetS following particulate inhalation exposures.

## Materials and methods

### AgNP characterization

Twenty nm silver nanoparticles (AgNPs) coated with citrate were purchased from NanoComposix (San Diego, CA, United States) suspended in water at a concentration of 1 mg/ml. AgNPs at a concentration of 25 µg/mL in DI water were characterized by assessment of hydrodynamic size, polydispersion, and ζ-potential via ZetaSizer (ZetaSizer Nano, Malvern) to verify manufacturer's specifications (n = 4).

### Animal models, diet-induced metabolic syndrome, resolvin D1 (RvD1) treatment, and AgNP exposure

C57BL/6 J male mice, at age of 6 weeks, obtained from Jackson Labs, were fed either a healthy or high-fat western (HFW) diet for 14 weeks. The healthy diet contained 10% of kcal fat and 51.6 mg/kg cholesterol (D12450B, Research Diets Inc., New Brunswick, NJ, United States), and the HFW diet contained 60% of kcal fat and 279.6 mg/kg cholesterol (D12492, Research Diets Inc., New Brunswick, NJ, United States). Animals were provided with water ad libitum and food was refreshed every other day. This HFW diet is well established to generate mouse models of MetS and has been used by our group and others for studies evaluating MetS [11–13, 78]. Specifically, other studies have demonstrated mice on this diet exhibit key factors of MetS such as obesity, elevated cholesterol, increased glucose, increased glucose tolerance, and hypertension [11–13, 30, 31, 75]. Males were included to allow for comparisons to previous studies establishing dysregulation of SPMs 24 h following AgNP exposure. Additionally, male mice demonstrate a greater differential in terms of disease (weight gain and dyslipidemia) compared to females on the HFW diet making them the initial model to use to investigate disease and lipid signaling [30, 79, 80]. Subsets of mice were exposed to either 50 µl of water (control) or 50 µg of 20 nm AgNP via oropharyngeal aspiration (50 µL of stock AgNPs at a concentration of 1 mg/mL in water). Twenty-four hours later, subsets of mice were treated with either 40 µl saline (control) or 400 ng of RvD1 in 40 µl saline (100 µg/mL) via oropharyngeal aspiration. Water was utilized as the control for AgNP exposure due to the AgNPs being commercially produced and suspended in water whereas saline was utilized as the control for the treatment because RvD1 has improved solubility in saline. This exposure and treatment protocol resulted in the generation of eight groups: (1) Healthy-Control-PBS, (2) Healthy-Control-RvD1, (3) Healthy-AgNP-PBS, (4) Healthy-AgNP-RvD1, (5) MetS-Control-PBS, (6) MetS -Control-RvD1, (7) MetS -AgNP-PBS, and (8). Subsets of mice were euthanized, and samples collected to assess markers of inflammation at 3, 7, 14, or 21 days following the AgNP exposure. The timepoints were selected based on previous studies examining pulmonary neutrophilic influx as an inflammatory endpoint following exposure to similar doses of AgNPs via oropharyngeal aspiration. Our previous studies demonstrated the differential induction of inflammatory markers (neutrophilic influx, inflammatory cytokines/chemokines) and reduction of SPMs at 24 h post-exposure [11, 13]. While other studies have demonstrated pulmonary neutrophil counts peaking at 3 days post-exposure and returning to baseline by 21 days post-exposure [27, 38, 81]. The AgNP dose of 50 μg/mouse was selected based on previous studies demonstrating the induction of an acute inflammatory response that can be used to study differences due to MetS and/or lipid interventions [81, 82]. 50 µg AgNP/mouse has been reported to stimulate inflammation characterized by pulmonary neutrophil cell influx and elevated mRNA expression of inflammatory markers in mouse models [11, 13, 82]. Further, a comparable dose in a healthy rat model demonstrated alterations in inflammatory markers which decreased in a time dependent manner with elevations still present at 21 days post-exposure [38]. 50 μg/mouse was also previously determined by our group to induce differential inflammation in the MetS model which corresponded with decreased pulmonary levels of RvD1 and other SPMs at 24 h post-exposure [11, 13]. An assessment of a AgNP manufacturing facility determined a mass concentration of 288 μg/m^3^ in the injection room [83]. Using this measurement, 50 μg/mouse would be equivalent to 180 days of human exposure. 400 ng of RvD1 in 40 µl saline has been previously utilized to attenuate acute inflammation [23, 84]. Prior to our experiment, we performed a small pilot study evaluating RvD1 treatment concentrations and timepoints post AgNP exposure in MetS mice to modulate inflammatory effects. This pilot study demonstrated 400 ng of RvD1 provided at 24 h post AgNP exposure was sufficient to reduce neutrophilic influx (data not shown). Additionally, a similar dosage was reported by other groups to reduce neutrophil recruitment, suppress proinflammatory markers and elevate the pro-resolution in inflamed lung mice triggered by acute cigarette smoke [24]. Lastly, our previous experiments have demonstrated reductions in SPMs including RvD1 at 24 h following AgNP exposure in the MetS mouse model while other studies observed peaking of SPMs between 24 and 48 h following exposure or challenge [11, 13, 63, 85, 86]. All animal procedures were conducted in accordance with the National Institutes of Health guidelines and approved by the Purdue University Animal Care and Use Committee.

### Model characterization

Body weight was measured before necropsy to determine alteration in body weight gain due to diet, exposure, and/or lipid treatment over the study time course. At necropsy, blood was collected via cardiac puncture and serum was isolated by centrifugation for quantification of circulating lipid levels. Specifically, total cholesterol, high-density lipoprotein (HDL), low-density lipoprotein (LDL) (Bioassay Systems, Hayward, California, United States), and triacylglycerides (Cayman Chemical, Ann Arbor, Michigan, United States) were assessed using commercially available kits following manufacturer's protocols.

### Collection of bronchoalveolar lavage fluid (BAL fluid)

BAL fluid was collected from the right lung to assess the alterations in cytology (i.e. influx of immune cells) within the lungs in response to AgNP exposure. BAL fluid has been utilized to evaluate the inflammatory response in the lungs in many previous studies [87]. Briefly, to collect BAL fluid, the left lung was tied off and the trachea was cannulated with a 20-gauge sterile syringe catheter. BAL fluid was collected from the right lung by gently washing it four times with individual volumes of cold phosphate buffer saline (PBS) (17.5 mL/kg body weight). The first lavage was collected, centrifuged (1800 rpm, 6 min, 4 °C), and the protein-rich supernatant, was stored at − 80 °C for total protein, inflammatory cytokines/chemokines evaluations. Total protein was measured using the BCA assay (Thermo Scientific, Hercules, CA, United States). Inflammatory cytokines and chemokines were measured within BAL fluid with ELISA (described below). BAL fluid pellets from all four washes were resuspended in PBS, combined, and counted using a cellometer (Nexcelom, MA, United States) to determine total cell counts. An equal number of cells were adhered to microscope slide before staining with a three-step hematology statin (Fisher Scientific, Newington, NH, United States) utilizing a Cytospin IV (Shandon Scientific Ltd., Cheshire, United Kingdom). Bright-field microscopy was utilized to view slides and cell types were identified and counted based on cellular morphology. At least 300 cells were counted per slide. Slides were counted blindly by two different individuals. The average of the cell counts were used to produce percentages of specific cell types within each BAL fluid sample and applied to the total cell counts to quantify individual cells in each sample. These assessments have been used previously in studies by our lab and other groups to examine acute lung inflammation and injury to the alveolar capillary barrier [11, 13, 88, 89].

### Hyperspectral imaging assessment of AgNPs

BAL fluid cell slides were also viewed and examined by enhanced hyperspectral dark-field microscopy (Cytoviva, Auburn, AL, USA), similar to previous assessments [13, 33, 34]. 2 μL of AgNPs (1 mg/mL) were pipetted directly onto a premium clean microscope slide and a mean spectrum was created using pixels with an intensity of > 1000 arbitrary units. This mean spectrum was then compared to AgNPs identified within BAL fluid cells to determine alterations following internalization. AgNPs within cells were selected by focusing on the nucleus of the cell, and a hyperspectral image was collected at a magnification of 100X. At least, 1000 pixels of internalized AgNPs were collected to form a mean spectrum. All spectra were then normalized and compared to the original spectrum of the AgNP sample. This assessment allowed for qualitative examination of AgNP uptake by BAL fluid cells as well as evaluation of spectral alterations due to internalization.

### mRNA expression analysis

Genes involved in inflammatory response and in resolution pathways were assessed from the left lung tissues using rtPCR. Briefly, 10 mg of lung tissue was placed in 2 mL vials containing 1.4 mm ceramic (zirconium oxide) beads (CK 14 soft tissue homogenizer Precellys, Bertin Technologies, Rockville, MD) and Trizol (Invitrogen, Carlsbad, CA, United States) and homogenized at a speed of 5 m/s for 30 s. Total RNA was extracted from the homogenate using Direct-zolTM RNA MiniPrep kits (Zymo Research, Irvine, CA, United States) in accordance with the manufacturer's instructions. A Nanodrop (Thermo Scientific, Hercules, CA, United States) was used to quantify RNA concentrations and evaluate quality. An aliquot of 1 µg of RNA was reverse transcribed into cDNA using an iScriptTM cDNA Synthesis Kit (Bio-Rad, Hercules, CA, United States) in accordance with the manufacturer's instructions. Quantitative real time rt-PCR analysis was performed using inventoried primers (Qiagen, Hidden, Germany) to evaluate altered gene expression of *interleukin-6 (IL-6), macrophage inflammatory protein-2 (MIP-2), monocyte chemoattractant protein-1 (MCP-1), tumor necrosis factor alpha (TNF-α), interleukin-10 (IL-10), interleukin-4 (IL-4), arachidonate 5-lipoxygenase (ALOX-5)*, *arachidonate 15-lipoxygenase (ALOX-15), inducible nitric oxide synthase (iNOS), and phospholipase A2 (iPLA2)*. In all altered gene expressions, *glyceraldehyde 3-phosphate dehydroge-nase (GAPDH)* was used as the internal control. Fold changes were calculated by comparing all samples values individually to the average of the time-matched control healthy mouse (healthy mouse group exposed to water and treated with saline).

### ELISA assays to evaluate BAL fluid cytokine and resolvin D1 levels

Cytokines and chemokines levels including macrophage inflammatory protein-2 (MIP-2), monocyte chemoattractant protein-1 (MCP-1), and interleukin-10 (IL-10) were quantified from collected BAL fluid using Mouse DuoSet ELISA kits (R&D Systems, Minneapolis, MN, United States). Resolvin D1 (RvD1) was also quantified from collected BAL fluid utilizing Cayman ELISA kit (Cayman Chemical, Ann Arbor, MI, United States).

### Statistical analysis

Results are expressed as mean values ± S.E.M. with five animals/control groups and eight animals/exposed groups. All samples were assessed in duplicate and values averaged for endpoints including serum parameters, BAL fluid cell counts, mRNA expression, and BALF cytokines and chemokines. For statistical analysis, a three-way analysis of variance (ANOVA) was used to determine statistical differences between groups at each distinct time point, with disease (healthy or MetS), exposure (control or AgNPs exposure), and treatment (RvD1 treatment or no RvD1 treatment), as the three factors. To examine time, a one-way Anova was performed comparing each group as defined by disease, exposure, and lipid treatment across time points evaluated. Bonferroni test was utilized for multi-comparison analysis. All statistical examinations were performed using GraphPad Prism 9 software (Graph Pad, San Diego, CA, United States), and *p* < 0.05 was considered to be statistically significant.

## Supplementary Information

**Additional file 1. Supplemental Figure 1. **Characterization of healthy and MetS mouse models.** Supplemental Figure 2. **Darkfield microscopy assessments of AgNPs within macrophages collected from BAL fluid from healthy and MetS mouse models not receiving RvD1 treatment.** Supplemental Figure 3. **Darkfield microscopy assessment of AgNPs within macrophages collected from BAL fluid from healthy and MetS mouse models receiving RvD1 treatment.** Supplemental Figure 4. **Hyperspectral analysis of AgNPs within neutrophils and macrophages collected in BAL fluid from health and MetS mouse models.

## Data Availability

The data will be made available according to the National Institutes of Health policies.

## Abbreviations

MetS: Metabolic syndrome
SPMs: Specialized pro-resolving lipid mediators
RvD1: Resolvin D1
AgNP: Silver nanoparticle
PM: Particulate matter
LMI: Lipid mediators of inflammatory
EPA: Eicosapentaenoic acid
DHA: Docosapentaenoic acid
18-HEPE: 18-Hydroxy eicosapentaenoic acid
14-HDHA: 14-Hydroxy docosahexaenoic acid
7-HDHA: 17-Hydroxy docosahexaenoic acid
MetS: Metabolic syndrome
SPMs: Specialized pro-resolving lipid mediators
RvD1: Resolvin D1
AgNP: Silver nanoparticle
PM: Particulate matter
LMI: Lipid mediators of inflammatory
EPA: Eicosapentaenoic acid
DHA: Docosapentaenoic acid
18-HEPE: 18-Hydroxy eicosapentaenoic acid
14-HDHA: 14-Hydroxy docosahexaenoic acid
7-HDHA: 17-Hydroxy docosahexaenoic acid
LPS: Lipopolysaccharides
BW: Body weight
TC: Total cholesterol
HDL: High-density lipoprotein
LDL: Low-density lipoprotein
TG: Triacylglycerides
HFW: High-fat western
BAL: Bronchoalveolar lavage
IL-6: Interleukin-6
MIP-2: Macrophage inflammatory protein-2
MCP-1: Monocyte chemoattractant protein-1
TNF-α: Tumor necrosis factor alpha
(NF-κB): Nuclear factor kappa-light-chain-enhancer of activated B cells
IL-10: Interleukin-10
IL-4: Interleukin-4
ALOX-5: Arachidonate 5-lipoxygenase
ALOX-15: Arachidonate 15-lipoxygenase
iNOS: Inducible nitric oxide synthase
iPLA2: Phospholipase A2
NPD1: Neuroprotectin 1
GAPDH: Glyceraldehyde 3-phosphate dehydrogenase
