# Supplementary material for: Enhanced silver nanoparticle-induced pulmonary inflammation in a metabolic syndrome mouse model and resolvin D1 treatment
Source: Part Fibre Toxicol. 2022 Aug 6;19:54. doi: 10.1186/s12989-022-00495-6 (PMC9356467; doi:10.1186/s12989-022-00495-6)
Supplement: Supplementary file 1 — Additional file 1. Supplemental Figure 1. Characterization of healthy and MetS mouse models. Supplemental Figure 2. Darkfield microscopy assessments of AgNPs within macrophages collected from BAL fluid from healthy and MetS mouse models not receiving RvD1 treatment. Supplemental Figure 3. Darkfield microscopy assessment of AgNPs within macrophages collected from BAL fluid from healthy and MetS mouse models receiving RvD1 treatment. Supplemental Figure 4. Hyperspectral analysis of AgNPs within neutrophils and macrophages collected in BAL fluid from health and MetS mouse models. [file 12989_2022_495_MOESM1_ESM.docx]

**Supplemental Figures**

**Supplemental Figure 1.**

**
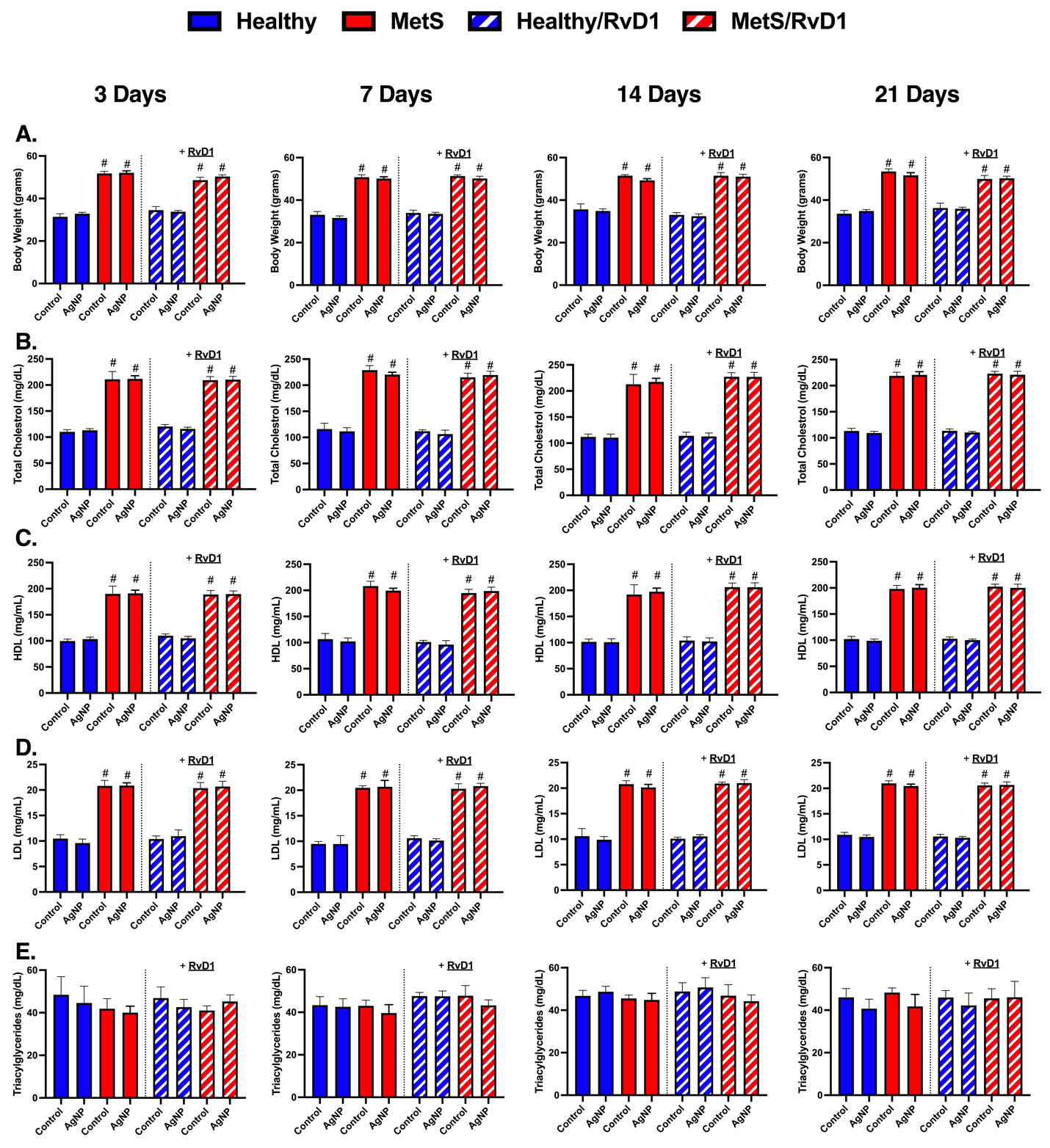
**

**Figure S1.** Characterization of body weight and serum lipid levels in healthy and MetS mouse models following 14 weeks on either a healthy or high-fat western diet (HFW Diet). (A) Body weight, (B) Total Cholesterol, (C) High-density lipoprotein, (D) Low-density lipoprotein, and (E) Triacylglyceride levels were evaluated in healthy and MetS mouse model serum at 3, 7, 14 and 21 days after oropharyngeal aspiration exposure to 50µg of AgNPs. A subset received sterile saline (vehicle) or 400 ng of RvD1 24 h post exposure. Values are expressed as mean ± S.E.M. * AgNP exposure; # disease model; $ treatment; and t time point (p<0.05).

**Supplemental Figure 2.**


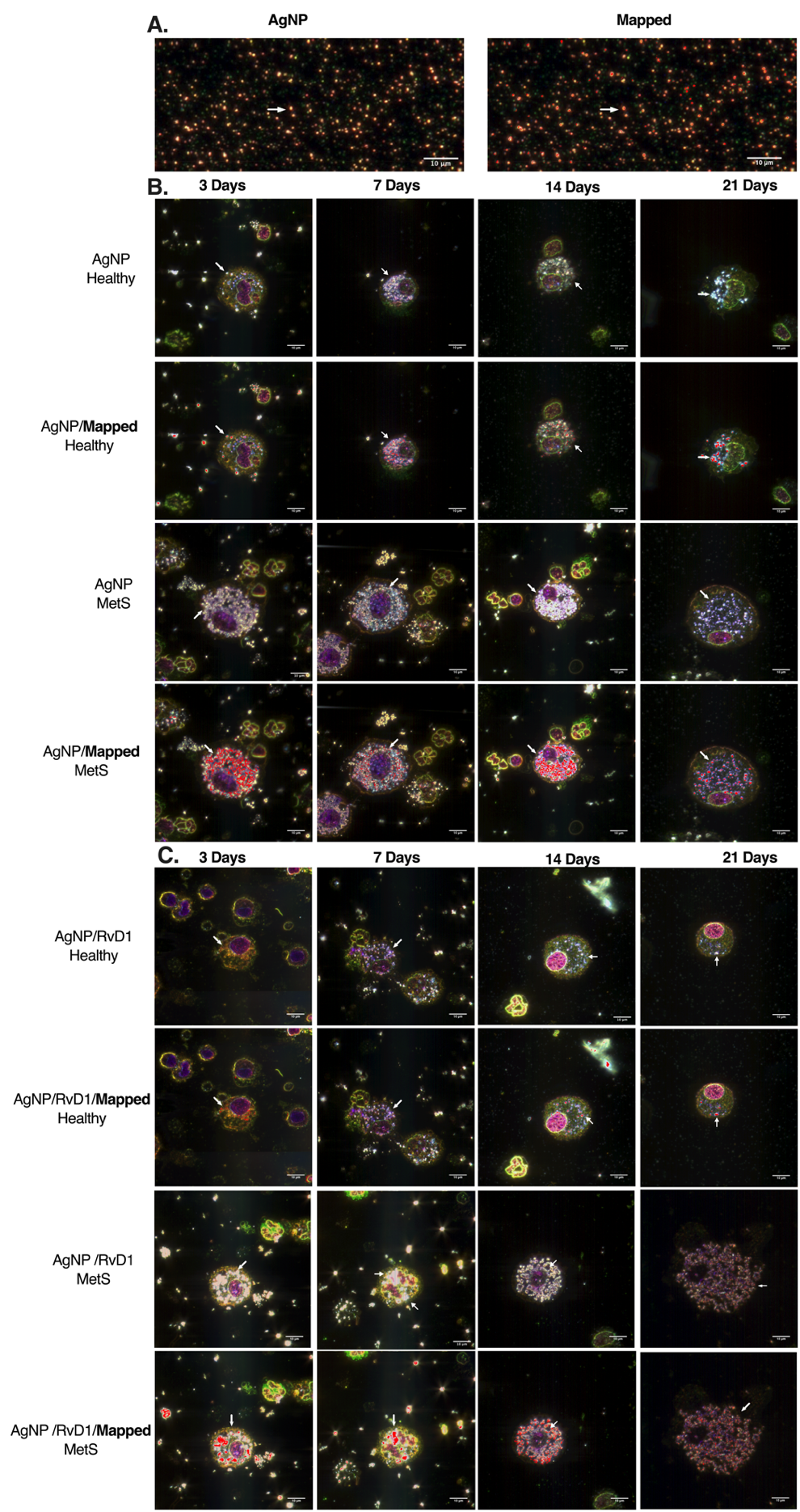


**Figure S2.** Darkfield microscopy assessment of of AgNPs within macrophages collected from BAL fluid healthy and MetS mouse models. (A) Representative darkfield images of original AgNP generated from over 1,000 pixels. (B) Representative enhanced darkfield images of macrophages at 3, 7, 14, and 21 days following AgNP exposure at 50 µg from healthy and MetS mice not receiving RvD1 treatment. (C) Representative enhanced darkfield images of macrophages at 3, 7, 14, and 21 days following AgNP exposure from healthy and MetS mice receiving 400 ng RvD1 treatment. White arrows indicate the accumulation of AgNPs within BAL fluid cells. White bar identifies 10 µm scaling. At least 1000 pixels of AgNPs were collected from mean spectra and then all spectra were normalized based on intensity for comparisons. These profiles were then mapped against representative images identifying only AgNPs in samples, confirming the accuracy of the spectral assessments.

**Supplemental Figure 3.**


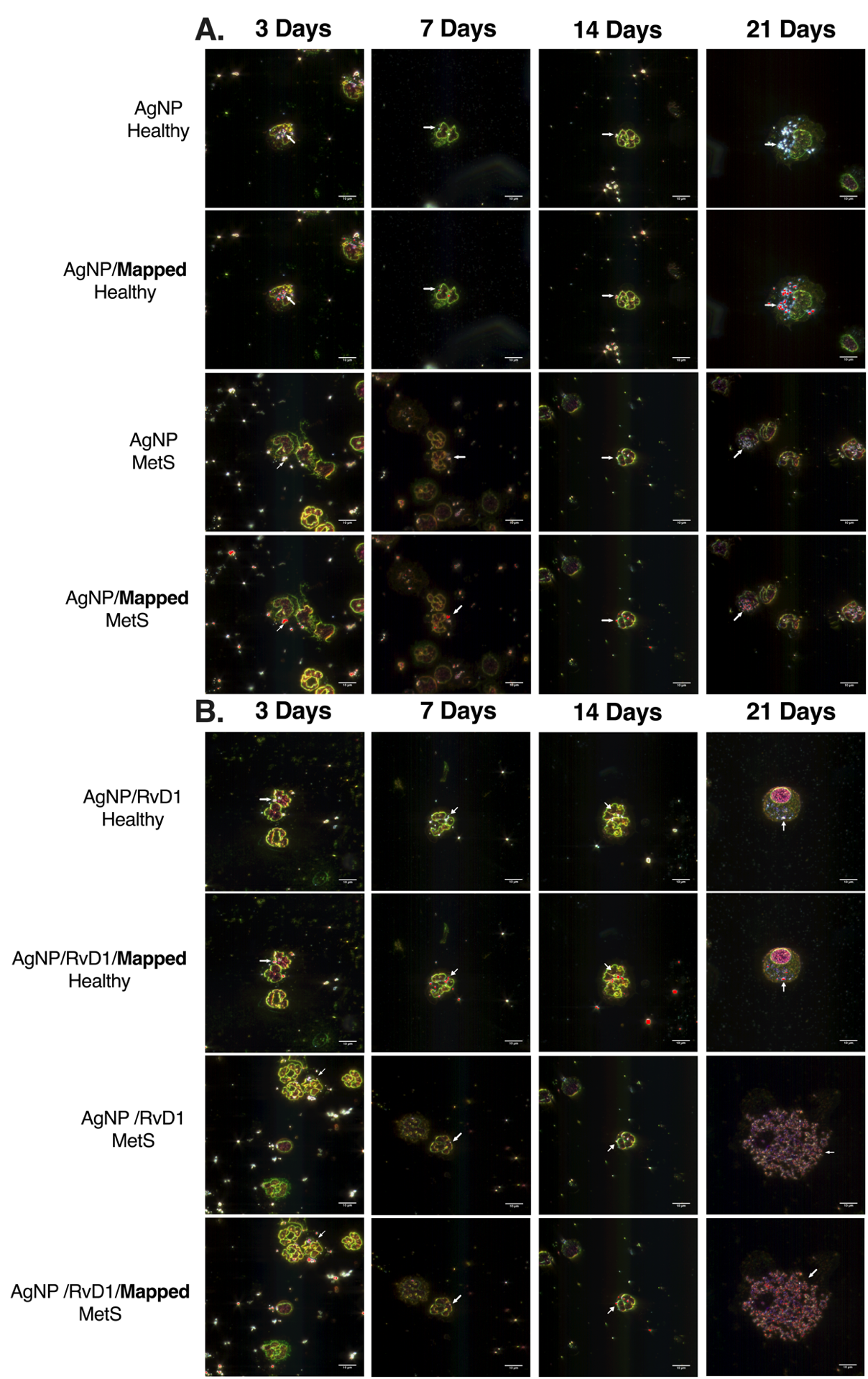


**Figure S3.** Darkfield microscopy assessment of AgNPs within neutrophil collected from BAL fluid healthy and MetS mouse models. (A) Representative enhanced darkfield images of neutrophil at 3, 7, 14, and 21 days following AgNP exposure at 50 µg from healthy and MetS mice not receiving RvD1 treatment. (B) Representative enhanced darkfield images of neutrophil at 3, 7, 14, and 21 days following AgNP exposure from healthy and MetS mice receiving 400 ng RvD1 treatment. Neutrophils were not observed in exposed healthy receiving and in not receiving RvD1 treatment at day 21. Neutrophils were not seen in exposed MetS receiving RvD1 treatment at day 21 days. White arrows indicate the accumulation of AgNPs within BAL fluid cells. At least 1000 pixels of AgNPs were collected from mean spectra and then all spectra were normalized based on intensity for comparisons. White bar identifies 10 µm scaling. These profiles were then mapped against representative images identifying only AgNPs in samples, confirming the accuracy of the spectral assessments.

**Supplemental Figure 4.**


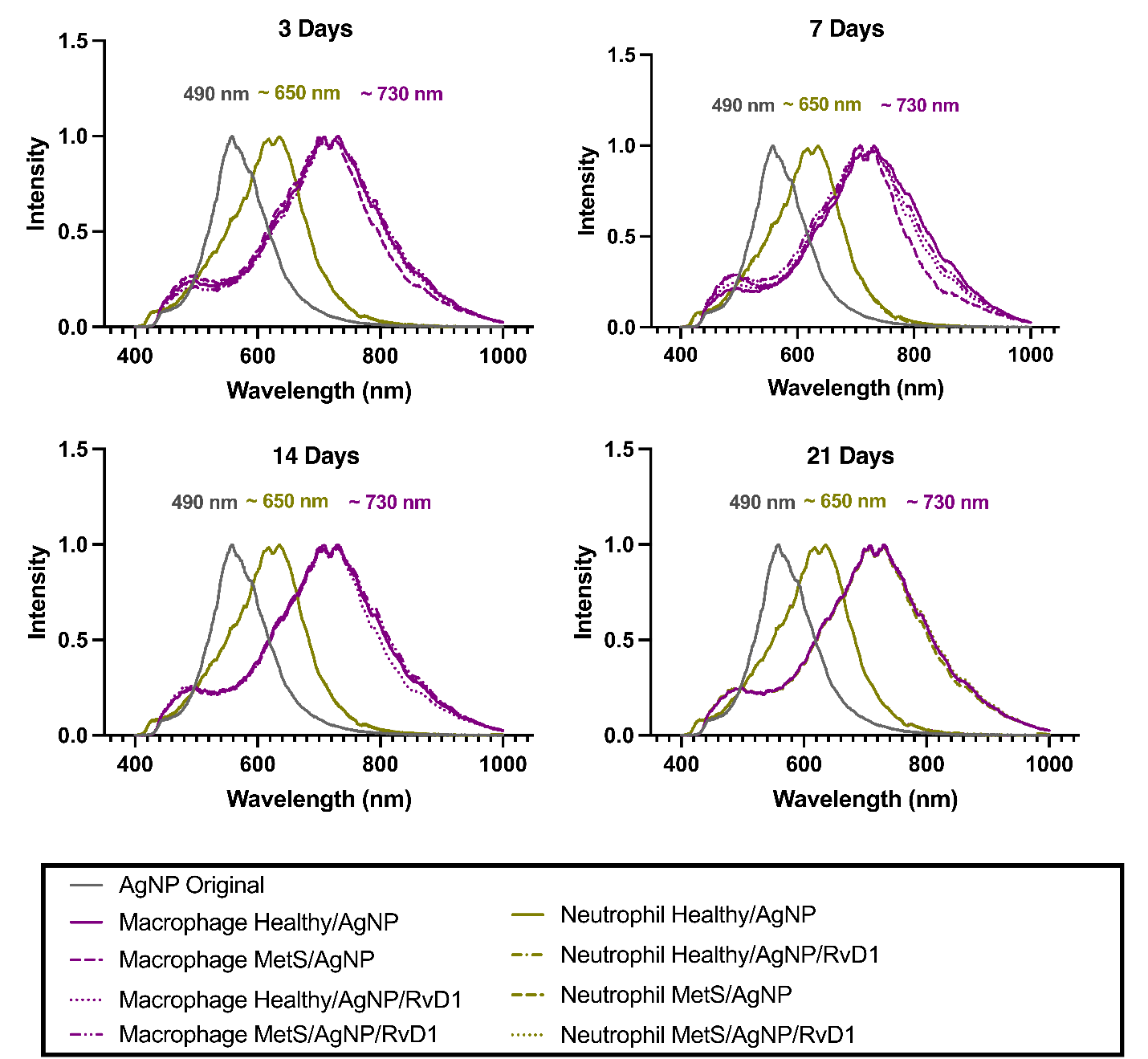


**Figure S4.** Hyperspectral analysis characterizing differences in mean spectra of 20 nm AgNPs within neutrophils and macrophages collected from BAL fluid healthy and MetS mouse models. Differences in mean spectra of AgNPs at 3, 7, 14, and 21 days following AgNP exposure at 50 µg from healthy and MetS mice receiving or/not receiving 400 µg RvD1 treatment. Gray curve indicates the spectral profile of the original AgNP sample, the green curve represents AgNPs internalized by neutrophils, and the purple curve represents AgNPs internalized by macrophages. Numbers correspond to peak wavelengths.
